# Homicidios en jóvenes y desigualdades sociales en México, 2017

**DOI:** 10.26633/RPSP.2019.94

**Published:** 2019-12-06

**Authors:** Oswaldo Sinoe Medina Gómez, Beatriz Villegas Lara

**Affiliations:** 1 Unidad de Investigación en Epidemiología Clínica Instituto Mexicano del Seguro Social Ciudad de México México Unidad de Investigación en Epidemiología Clínica, Instituto Mexicano del Seguro Social, Ciudad de México, México.; 2 Universidad Autónoma Metropolitana Xochimilco Universidad Autónoma Metropolitana Xochimilco Ciudad de México México Universidad Autónoma Metropolitana Xochimilco, Ciudad de México, México.

**Keywords:** Homicidio, determinantes sociales de la salud, disparidades en el estado de salud, México, Homicide, social determinants of health, health status disparities, Mexico, Homicídio, determinantes sociais da saúde, disparidades nos níveis de saúde, México

## Abstract

**Objetivo.:**

Conocer la asociación entre las condiciones sociales y económicas y las tasas de homicidios en jóvenes de 10 a 24 años de edad en México en 2017.

**Métodos.:**

En este estudio ecológico se estudiaron las desigualdades sociales asociadas con los homicidios en la población de 10 a 24 años en 2017 en México a través de fuentes secundarias de información, correspondientes a las defunciones por homicidio por entidad federativa en México. Las desigualdades sociales en salud se estudiaron mediante la medición absoluta y relativa de las brechas de desigualdad y se estimaron razones de prevalencias de mortalidad con modelos de regresión de Poisson.

**Resultados.:**

En 2017 se produjeron 8 094 homicidios en la población joven, que fueron más frecuentes en hombres (86,7%). Las brechas de desigualdad entre los estados fueron importantes. La desocupación de la población de mayores de 12 años, los hogares conformados por personas que no son familiares, la baja asistencia escolar y el ingreso por debajo de la línea de bienestar se asociaron de manera significativa con las tasas de homicidios.

**Conclusiones.:**

La asociación de los determinantes sociales con los homicidios en la población estudiada es fuerte. Deben implementarse políticas y acciones intersectoriales que puedan ayudar a reducir las brechas de desigualdad y lograr mejores condiciones de vida y niveles de bienestar y salud de las personas y sus comunidades.

## Abstract (en)

**Objective.:**

Assess the association between social and economic conditions and homicide rates in young people between 10 and 24 years of age in Mexico in 2017.

**Methods.:**

This ecological study looked at the social inequalities associated with homicides in the population 10-24-year-old population in 2017 in Mexico, using secondary data sources for deaths by homicide in each Mexican state. Social inequalities in health were studied by measuring absolute and relative inequality gaps. Mortality rates were estimated using Poisson regression models.

**Results.:**

In 2017, there were 8,094 homicides in the target population, predominantly in men (86.7%). There was high variance in inequality between states. Unemployment in the population over 12 years of age, households composed of people who are not family members, low school attendance rates, and income below the poverty line showed significant association with homicide rates.

**Conclusions.:**

There is a strong association between social determinants and homicides in the study population. Policies and intersectoral actions should be implemented to help bridge inequality gaps and achieve better living conditions and higher levels of well-being and health for people and their communities.

## RESUMO

**Objetivo.:**

Descrever a associação entre as condições socioeconômicas e as taxas de homicídios em jovens entre 10 e 24 anos no México em 2017.

**Métodos.:**

Neste estudo ecológico foram examinadas as desigualdades sociais associadas aos homicídios na população entre 10 e 24 anos no México em 2017 com base em dados obtidos de fontes de informação secundárias relativos a mortes por homicídio por entidade federativa. As desigualdades sociais em saúde foram avaliadas com a mensuração absoluta e relativa das lacunas de desigualdade. As razões de prevalências de mortalidade foram estimadas com modelos de regressão de Poisson.

**Resultados.:**

Em 2017, houve 8.094 homicídios na população jovem, com predomínio no sexo masculino (86,7%). As lacunas de desigualdade entre os estados foram significativas. Ociosidade juvenil (em maiores de 12 anos), domicílios constituídos por pessoas sem laços de parentesco, baixa frequência escolar e renda abaixo da linha do bem-estar foram fatores que tiveram uma associação significativa com as taxas de homicídios.

**Conclusões.:**

Observou-se uma forte associação entre os determinantes sociais e os homicídios na população estudada. Políticas e ações intersetoriais devem ser implementadas para ajudar a reduzir as lacunas de desigualdade e melhorar as condições de vida, o nível de bem-estar e a saúde das pessoas e suas comunidades.

La violencia, dada sus características y consecuencias complejas, se considera uno de los principales problemas de salud pública a nivel mundial (1,2). Tiene relevancia política, porque aumenta cuando no existe democracia, respeto por los derechos humanos ni condiciones de buen gobierno (3).

El homicidio, como expresión final de los diversos tipos de violencia, ha ganado poca atención de los investigadores de salud pública y los responsables de la política de salud, a pesar de ser una causa de muerte prevenible, y el análisis de sus datos y distribución pueden aportar indicios del grado de violencia mortal en un país o entidad federativa determinada (3-5).

Según el Programa de las Naciones Unidas para el Desarrollo (PNUD), América Latina se ha convertido durante la última década en la región más insegura del mundo, con un incremento constante de la tasa de homicidios y un desempeño menor de lo esperado por parte de sus países para disminuir las tasas de mortalidad por esta causa (6).

Las defunciones por homicidios en América Latina son más frecuentes en hombres que en mujeres, y la tasa de homicidios de jóvenes –alrededor de 70 por 100 000 jóvenes– es superior al doble de la tasa en toda la población (7).

En México, la tasa de homicidios en la población general disminuyó entre 1992 y 2007, pero en los dos años posteriores se duplicó (de 7,6 a 16,6 por 100 000). En 2009, la tasa de homicidios en hombres fue cerca de 9 veces mayor que la tasa en mujeres y casi dos tercios de los homicidios fueron por arma de fuego (8). Se ha demostrado que el incremento de los homicidios en México se relaciona, principalmente en los hombres, con la ofensiva implementada contra las organizaciones de traficantes de drogas (9), a diferencia de las mujeres, que se asocia principalmente a condiciones socioeconómicas y culturales (10). Dicho incremento en los homicidios ha tenido repercusiones en el retroceso de la esperanza de vida en los hombres (11).

Es importante reconocer que los problemas de salud requieren una aproximación transdisciplinaria que permita identificar el proceso salud-enfermedad como un proceso histórico-social, para develar el origen de las expresiones diferenciadas e inequitativas en la mortalidad de los grupos sociales y reconocer la interacción entre las distintas dimensiones biológicas, sociales y económicas (12). Algunos autores han subrayado la asociación entre los homicidios en los jóvenes y las condiciones socioeconómicas, como las características del vecindario, el nivel socioeconómico y las oportunidades educativas y culturales (13-15).

Por todo ello, con el presente estudio se pretende conocer la asociación entre las condiciones sociales y económicas y las tasas de homicidios en jóvenes de 10 a 24 años de edad durante 2017 en México.

## MATERIALES Y MÉTODOS

Se realizó un estudio ecológico a partir de fuentes secundarias de información correspondientes a las defunciones ocurridas durante 2017 en la población de 10 a 24 años, identificada como población joven según la Organización Mundial de la Salud (OMS) (16), en cada entidad federativa en México donde se produjo el homicidio. El Instituto Nacional de Estadística y Geografía (INEGI) fue la fuente de información para conocer el número de defunciones a nivel nacional y por entidad federativa. Los datos se obtuvieron sobre la base de la Clasificación Internacional de Enfermedades 10a edición (CIE-10), donde los homicidios aparecen en el rango de códigos X85 a Y09.

Para el análisis, se excluyeron las defunciones que se produjeron en el extranjero, así como aquellas con registro de edad no especificada. Como mecanismo de control, para identificar un posible subregistro en la mortalidad, se midió el número de casos con diagnóstico de eventos de intención no determinada bajo los códigos Y10-Y34, así como el código Y87.1 (secuelas de agresión), que se integraron al registro total de homicidios. Los datos obtenidos correspondieron a la entidad federativa donde tuvo lugar el homicidio. No se consideró redistribuir los casos de homicidios con edad no especificada en la población de estudio, porque el análisis por grupos de edad no tenía incidencia alguna en los resultados.

La tasa de mortalidad general por homicidios se definió como el número de defunciones dividido por el total de población en un año determinado según la población estimada por el Consejo Nacional de Población (CONAPO) por cada 100 000 habitantes de 10 a 24 años (17). Se calcularon tasas específicas para los grupos de edad de 10 a 14 años, de 15 a 19, y 20 a 24 años, así como la tasa específica de homicidios por sexo.

Para analizar los datos, se utilizó el paquete estadístico Stata versión 12,0. El análisis descriptivo se realizó con cada indicador socioeconómico de cada entidad federativa y se calculó la tasa de homicidios para cada uno de los grupos de edad señalados previamente. Para conocer las brechas de desigualdad entre las entidades federativas, se calcularon medidas absolutas y relativas para valores no ordenados propuestas por la OMS (cuadro 1) con el programa Health Equity Assessment Toolkit Plus (HEAT Plus) 2,0 (18,19).

**CUADRO 1.** Medidas de desigualdad propuestas por la OMS

Tipo de medida	Medida de desigualdad	Fórmula	Interpretación
Absolutas			
	Desviación estándar entre grupos	$BGSD=\;{\sqrt{\sum }}_{J}P_{J}{\left(YI-\mu \right)}^{2}$	BGSD es cero si no hay desigualdad
	Varianza entre grupos	$BGV=\sum_{i}{\left(y_{j}-\mu \right)}^{2}$	Cuanto mayor es el valor positivo, mayor es el nivel de desigualdad
	Riesgo atribuible poblacional	$PAR=y_{ref-\mu }$	Cuanto mayor es el valor, mayor es el nivel de desigualdad
	Diferencia	D=yhigh−ylow	Cuanto mayor es el valor absoluto, mayor es el nivel de desigualdad
	Diferencia media ponderada del promedio	MDMW=∑j pj |yf−μ|	MDMW es cero si no existe desigualdad
	Diferencia media ponderada del subgrupo de mejor rendimiento	$MDB=\;\sum^{\text{​}}j\;pj\left|yf-y\;ref\right|$	Valores más grandes indican niveles más altos de desigualdad
	Diferencia media no ponderada del promedio	$MDBU=\sum^{\text{​}}j\;\left|yf-y\;ref\right|$	MDBU es cero si no hay desigualdad
Relativas			
	Índice de disparidad ponderado	$IDIS\_W=\frac{\sum^{\text{​}}j\;Pj\left|yj-\;\mu \right|}{\mu }*100$	IDIS muestra valores mayores que indican niveles más altos de desigualdad
	Desviación log media	$MDL=\Sigma_{j}p_{j}(-\ln\;(y_{j}/\mu ))*1000$	Cuanto mayor es el valor absoluto de MLD, mayor es el nivel de desigualdad
	Coeficiente de variación	$COV=\frac{BGSD}{\mu }*100$	COV es cero si no hay desigualdad
	Fracción atribuible poblacional	$PAF=\frac{PAR}{\mu }*100$	PAF es cero si no se puede lograr una mejora adicional
	Índice de disparidad no ponderado	$IDISU=\frac{1}{n}*\frac{\sum^{\text{​}}j\;\left|yj-\;\mu \right|}{\mu }*100$	IDISU es cero si no hay desigualdad
	Razón	$R=y_{high}/y_{low}$	Cuanto mayor sea el valor de R de 1, mayor será el nivel de desigualdad
	Índice de Theil	$TI=\;\sum_{j\;p_{j\;}\;\frac{y_{j\;}}{\mu }}\ln\frac{y_{j}}{\mu }*1000$	Cuanto mayor es el valor absoluto de TI, mayor es el nivel de desigualdad

***Fuente:*** modificado a partir de la referencia 19.

Además, se analizaron las condiciones de vida de los adolescentes estudiando el número de hogares conformados por personas que no son familiares y están habitados por adolescentes de 12 a 19 años, la no asistencia escolar de la población mayor de 3 años, el ingreso inferior a la línea mínima de bienestar (descrito por el Consejo Nacional de Evaluación de la Política de Desarrollo Social (CONEVAL) como un ingreso inferior al valor de la canasta básica), y, finalmente, la población de 12 años y más que se encuentra desocupada. Los datos se obtuvieron para cada entidad federativa a partir de la encuesta intercensal 2015 del INEGI (20) excepto el ingreso inferior a la línea de bienestar, que se obtuvo a partir de la información publicada por el CONEVAL (21). Toda la información se recabó entre mayo y agosto de 2019.

Los valores de las variables sociales a nivel estatal se clasificaron en quintiles considerando al quintil 1 como valor de referencia del grupo con la mejor condición social. Para estimar la razón de prevalencias (RP) de mortalidad, se construyeron modelos de regresión de Poisson. Para ello, el número de homicidios en 2017 por cada grupo de edad, teniendo en cuenta las posibles diferencias entre los grupos de jóvenes, fue la variable dependiente, la población por grupo de edad en el mismo año se consideró variable de exposición, y las variables independientes fueron los indicadores sociales agrupados por quintiles a nivel de cada entidad federativa.

## RESULTADOS

En 2017 se registraron 8 094 homicidios de personas de 10 a 24 años; la tasa de homicidios por 100 000 personas de 10 a 24 años fue 24,43, 830 defunciones se clasificaron como eventos de intención no determinada y una defunción que correspondió a un hombre del estado de Sinaloa se registró como una defunción secuela de homicidio. El 86,7% de las víctimas fueron hombres y 62,7% de los homicidios fueron de personas de 20 a 24 años.

En 2017, Colima ocupó el primer lugar en homicidios (82,28 homicidios por 100 000 personas de 10 a 24 años), seguido de Campeche (81,04 homicidios por 100 000 personas de 10 a 24 años), mientras que Yucatán ocupó el último lugar a escala nacional (1,89 homicidios por 100 000 personas de 10 a 24 años).

**FIGURA 1.**
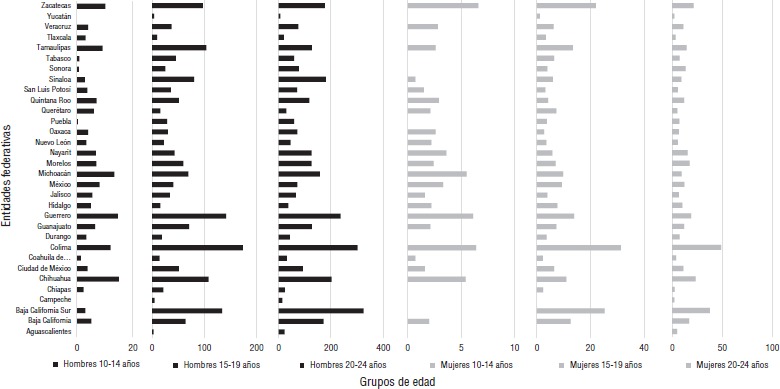
Tasas de homicidios por grupos de edad (x 100 000 personas) y entidad federativa, México 2017

Por grupos de edad, la tasa de homicidios específica para el grupo de 10 a 14 años a nivel nacional fue de 2,85 homicidios por 100 000 personas de 10 a 14 años; para el grupo de 15 a 19, la tasa específica por edad fue de 24,40 defunciones por 100 000 personas de 15 a 19 años, mientras que para el grupo de 20 a 24 años, fue de 46,58 homicidios por 100 000 personas de 20 a 24 años. Las tasas por entidad federativa y grupo de edad se presentan en la figura 1.

***Fuente:*** elaboración propia a partir de datos del Instituto Nacional de Estadística y Geografía, México, 2017.

**FIGURA 2.**
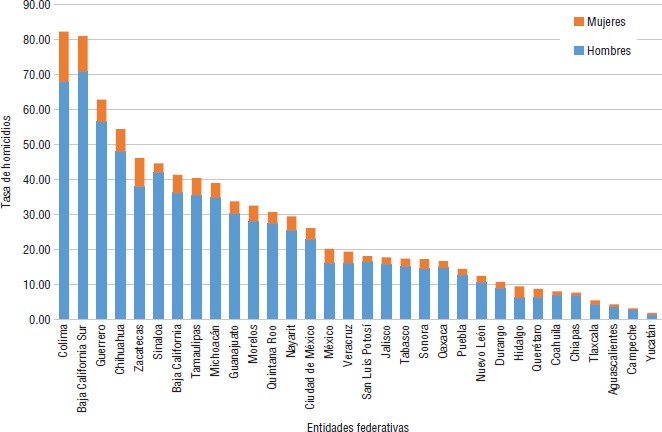
Tasas de homicidios por sexo en personas de 10 a 24 años (x 100 000 personas) y entidad federativa, México 2017

La tasa de homicidios específica por sexo de la población estudiada muestra que la entidad federativa con la mayor tasa de homicidios en hombres fue Baja California (70,91 homicidios por 100 000 hombres de 10 a 24 años), mientras que en las mujeres, la mayor tasa se registró en el estado de Colima (14,31) (Figura 2).

***Fuente:*** elaboración propia a partir de datos del Instituto Nacional de Estadística y Geografía, México, 2017.

Respecto a las mediciones de desigualdad, la diferencia de las tasas máximas y mínimas de homicidios en personas de 10 a 24 años por entidad federativa durante 2017 fue 80,38. El índice de disparidad ponderada obtenida fue 49,71, en tanto que tasas de mortalidad entre los estados muestran una varianza elevada (235,78).

La razón atribuible proporcional, que permite conocer las defunciones que podrían evitarse considerando la mortalidad del estado con menor tasa de homicidios, fue de -22,24. La desviación logarítmica de la media también refleja índices de desigualdad importantes al igual que los resultados obtenidos con el índice de Theil comparando las entidades federativas (186,10). Los resultados completos se encuentran en el cuadro 2.

**CUADRO 2.** Medidas de desigualdad en homicidios, México, 2017

Tipo de medidas	Medida de desigualdad	Medición entre entidades federativas
Absolutas		
	Desviación estándar entre grupos	15,36
	Varianza entre grupos	235,78
	Riesgo atribuible poblacional	-22,24
	Diferencia	80,38
	Diferencia media ponderada del promedio	12,00
	Diferencia media ponderada del subgrupo de mejor rendimiento	22,24
	Diferencia media no ponderada del promedio	527,75
Relativas		
	Índice de disparidad ponderado	49,71
	Desviación logarítmica media	208,88
	Coeficiente de variación	63,63
	Fracción atribuible poblacional	-92,15
	Índice de disparidad no ponderado	68,34
	Razón	43,43
	Índice de Theil	186,10

***Fuente:*** elaboración propia a partir de información del Instituto Nacional de Estadística y Geografía, México.

Los resultados de la regresión de Poisson muestran que la tasa de homicidios en las entidades federativas presenta un gradiente respecto a las condiciones de desocupación en todos los grupos de edad. La razón de prevalencias de mortalidad de mayor magnitud se presentó en el quintil 5 del grupo de 10 a 14 años ([RP = 4,42; IC95%: 2,27-8,60). Respecto a los hogares conformados por personas que no son familiares de los jóvenes, en el grupo de 10 a 19 años, la razón de prevalencias de mayor magnitud se encontró en el tercer quintil (RP = 3,31; IC95%: 1,80-6,09). Además, se encontró una asociación estadísticamente significativa en el quintil 5 de los grupos de 15 a 19 años (RP = 3,41; p < 0,001) y de 20 a 24 años (RP = 4,48; p < 0,001) (cuadro 3).

**CUADRO 3.** Asociación entre los homicidios en jóvenes y las condiciones socioeconómicas, México, 2017

Variables sociales	10 a 14 años				15 a 19 años				20 a 24 años			
	RP	P	IC95%		RP	P	IC95%		RP	P	IC95%	
Asistencia escolar en población mayor de 3 años												
Quintil 2	1,69	0,13	0,86	3,30	1,56	< 0,001	1,29	1,89	1,76	< 0,001	1,55	2,01
3	2,94	< 0,001	1,56	5,53	2,41	< 0,001	1,99	2,92	3,03	< 0,001	2,63	3,48
4	1,22	0,57	0,62	2,41	1,22	0,07	0,99	1,50	1,66	< 0,001	1,43	1,92
5	1,04	0,90	0,56	1,95	1,85	< 0,001	1,51	2,26	1,98	< 0,001	1,71	2,30
Hogares que no son familiares												
Quintil 2	2,47	0,01	1,29	4,73	1,59	< 0,001	1,30	1,95	1,83	< 0,001	1,58	2,12
3	3,31	< 0,001	1,80	6,09	2,17	< 0,001	1,83	2,59	2,24	< 0,001	1,97	2,55
4	2,78	< 0,001	1,47	5,27	2,23	< 0,001	1,83	2,71	2,47	< 0,001	2,15	2,85
5	2,49	0,04	1,03	6,01	3,41	< 0,001	2,66	4,36	4,48	< 0,001	3,76	5,34
Ingreso inferior a la línea mínima de bienestar												
Quintil 2	2,53	0,01	1,26	5,07	2,34	< 0,001	1,92	2,85	2,18	< 0,001	1,90	2,49
3	1,14	0,73	0,54	2,42	1,40	< 0,001	1,11	1,76	1,40	< 0,001	1,19	1,65
4	1,16	0,72	0,52	2,58	0,70	0,01	0,54	0,90	0,83	0,05	0,69	1,00
5	1,30	0,46	0,64	2,65	1,30	0,01	1,05	1,61	1,59	< 0,001	1,38	1,84
Población de 12 años y más desocupada												
Quintil 2	1,08	0,81	0,59	1,97	0,72	< 0,001	0,60	0,86	0,66	< 0,001	0,58	0,75
3	1,01	0,99	0,47	2,17	0,72	< 0,001	0,58	0,89	0,60	< 0,001	0,52	0,70
4	3,33	< 0,001	1,73	6,39	1,69	< 0,001	1,41	2,03	1,37	< 0,001	1,21	1,55
5	4,42	< 0,001	2,27	8,60	2,72	< 0,001	2,25	3,30	1,76	< 0,001	1,53	2,02

***Fuente:*** elaboración propia a partir de datos del Instituto Nacional de Estadística y Geografía, México.

RP = razón de prevalencias.

IC95%: intervalo de confianza del 95%.

Al considerar el ingreso inferior a la línea mínima de pobreza como variable proxy de marginación y condiciones de vida deplorables, la razón de prevalencias y estadísticamente significativa correspondió al quintil 2 de los grupos de edad de 10 a 14 años (RP = 2,53), de 15 a 19 años (RP = 2,34) y de 20 a 24 años (RP = 2,18).

Por otro lado, se observó que no acudir a centros escolares presenta una asociación importante respecto a los homicidios ocurridos en cada uno de los grupos de edad. Entre la población de 15 a 19 años, la magnitud de la razón de prevalencias de las entidades ubicadas en el segundo quintil fue dos veces mayor que la del grupo de referencia (RP = 2,41; p < 0,001), como las del grupo de 15 a 19 años (RP = 2,41; p < 0,001), y las de 20 a 24 años, tres veces mayor (RP = 3,03; p < 0,001) (cuadro 3).

## DISCUSIÓN

Dado el tipo de estudio realizado, debe tenerse en cuenta la posibilidad de incurrir en la falacia ecológica, sin que ello reste valor a los resultados obtenidos. Asimismo, hay limitaciones asociadas con el uso de fuentes de información secundarias para identificar las condiciones sociales que determinan las causas específicas de los homicidios en hombres y mujeres.

Por otro lado, es imperativo considerar la calidad del registro de los certificados de defunción vinculada con las condiciones en que se producen los homicidios, así como el subregistro del número de personas desaparecidas cuya situación se desconoce (22). Además, los registros de los certificados de defunción tienen limitaciones para estudiar los homicidios desde una perspectiva de género, por lo que es necesario integrar en el registro de las defunciones información acerca del sexo y el parentesco del homicida, entre otras variables que pudieran ser de interés.

Con relación a los resultados obtenidos, destacan las diferencias entre los estados y su relación con las tasas de homicidios. Las mediciones de desigualdad absolutas y relativas que se obtuvieron indican la existencia de grandes brechas de desigualdad entre las entidades federativas. Sin embargo, es necesario tener en cuenta la posibilidad de que estos resultados no reflejen el panorama real en cada estado, dado que existen municipios que concentran una mayor mortalidad por homicidios (25), lo cual subraya la necesidad de realizar estudios que tengan en cuenta el contexto específico de cada espacio geográfico.

Los resultados de este estudio muestran, además, que las mayores tasas de mortalidad se registran en los hombres jóvenes. Se ha señalado que este grupo de población tiene los mayores niveles mortalidad por homicidios porque los jóvenes carecen de oportunidades laborales y son más propensos a verse involucrados en actividades criminales (23). Los resultados también revelan que las condiciones de marginalidad y desocupación se asocian fuertemente con mayores niveles de homicidios en los grupos de edad estudiados, lo cual es similar a lo notificado en otro estudio (24).

Por otra parte, al analizar algunos de los determinantes sociales en su dimensión estatal, se identifican de forma congruente situaciones diferenciadas de vulnerabilidad social, desempleo y desarrollo humano. Estos determinantes se corresponden con desigualdades en las tasas de mortalidad por homicidio y se expresan como brechas en la mortalidad entre los estados, los niveles de marginalidad y de escolaridad.

Respecto a la composición familiar, se ha encontrado una asociación estadísticamente significativa entre los homicidios y la pertenencia a familias que están constituidas por personas que no son familiares de los jóvenes. Ello no debe considerarse un resultado concluyente, porque han de analizarse otros aspectos importantes, como la dinámica y la funcionalidad de las familias sin limitarse únicamente a la estructura familiar.

Para concluir cabe indicar que, ante la complejidad del problema y los resultados obtenidos, se considera necesario diseñar políticas sociales que permitan lograr el bienestar de las personas y sus familias, sobre todo si se reconoce la asociación entre los niveles de vulnerabilidad social, de desempleo y las brechas de desigualdad en el ingreso con una mayor tasa de homicidios. La implementación de políticas y de acciones intersectoriales podría ayudan a reducir las brechas de desigualdad y mejorar las condiciones de vida de los individuos y sus comunidades, y los niveles de bienestar y salud (26).

Además, es importante reconocer que los problemas de salud exigen una aproximación transdisciplinaria que permita identificar el proceso salud-enfermedad como un proceso histórico-social para develar el origen de las expresiones diferenciadas e inequitativas en la mortalidad de los grupos sociales y reconocer la interacción entre las distintas dimensiones biológicas, sociales y económicas (12,27,28).

Por último, cabe hacer hincapié en la importancia de medir las brechas de desigualdad como un mecanismo de diseño y evaluación de las políticas públicas, incluidas las políticas de salud en materia de promoción, prevención y atención.

## Contribución de los autores.

Los dos autores han participado en el diseño del estudio original, el análisis de los datos, la interpretación de los resultados y en la redacción, revisión y aprobación del manuscrito final.

## Financiación.

Los autores declaran no haber recibido ninguna financiación para realizar este estudio.

## Declaración.

Las opiniones expresadas en este manuscrito son responsabilidad de los autores y no reflejan necesariamente los criterios ni la política de la *RPSP/PAJPH* y/o de la OPS.
